# Metastatic Papillary Thyroid Carcinoma in the Lymph Nodes Without Identifiable Primary Tumor in the Thyroid

**DOI:** 10.7759/cureus.84554

**Published:** 2025-05-21

**Authors:** Amber Carrillo, Farinaz Arbab, Nisha S Ramani

**Affiliations:** 1 Department of Pathology and Immunology, Baylor College of Medicine, Houston, USA; 2 Department of Pathology and Immunology, Michael E. DeBakey Department of Veterans Affairs Medical Center, Houston, USA

**Keywords:** isolated metastasis, lymph node, metastatic, papillary thyroid carcinoma, thyroid

## Abstract

Papillary thyroid carcinoma (PTC) is the most common thyroid neoplasm. While its initial presentation as metastasis is not uncommon, metastasis to the lymph nodes without a primary tumor is extremely rare. Here we report an unusual case of metastatic PTC within the lymph nodes without an identifiable primary tumor in the thyroid gland.

We report a case of a 45-year-old man who presented with neck swelling detected on a routine physical examination. The patient was asymptomatic, and his thyroid function was normal. Ultrasound of the thyroid revealed a well-circumscribed isoechoic 4.5 cm mass in the mid/lower pole of the right lobe. The patient had a congenitally absent left thyroid lobe. CT neck showed no evidence of cervical lymphadenopathy. Fine-needle aspiration was suspicious for a neoplasm, and surgical excision was recommended. The patient then underwent right thyroid lobectomy and isthmusectomy. Gross examination of the thyroid revealed two nodules (4.4 cm and 2 cm) in the right lobe. The entire lobe and isthmus, including the two nodules, were submitted for histologic evaluation, which revealed two encapsulated lesions with microfollicular architecture. The cells displayed hyperchromatic small round cuboidal nuclei with absent nuclear features of PTC. These findings were consistent with follicular adenomas. Interestingly, two of the six central neck lymph nodes were positive for subcapsular metastatic PTC.

## Introduction

Papillary thyroid carcinoma (PTC) is the most common neoplasm in the thyroid, comprising about 80%-85% of cases, and it carries an excellent prognosis [1,2]. The incidence of PTC increases with radiation exposure, although genetics and diet can also play a role in the development of PTC [1]. The presentation of PTC is subtle, as most patients are often asymptomatic without any derangements in thyroid levels [1]. PTC is known for invading lymphatics; about 10% of cases can initially present with metastasis [1,3]. However, metastatic thyroid carcinoma without a primary tumor in the thyroid is rare [4]. We report a case of metastatic PTC to the lymph nodes without an identifiable primary tumor in the thyroid.

This article was previously presented as a poster at the 2024 Texas Society of Pathologists 103rd Annual Meeting on February 2, 2024.

## Case presentation

A 45-year-old man without significant past medical history presented to our care with a complaint of neck swelling. Physical examination revealed a mass in the right thyroid region with no palpable cervical lymphadenopathy. The patient had no other complaints, and thyroid-stimulating hormone was normal at 1.91 uIU/mL. Thyroid ultrasonography showed a well-circumscribed isoechoic solid mass occupying the mid and lower pole region of the right lobe, measuring 4.5 cm in greatest diameter. Notably, the patient had a congenitally absent left thyroid gland. Fine-needle aspiration of the right thyroid mass was performed, showing cells with a predominant microfollicular pattern and minimal colloid. It was called “suspicious for a neoplasm,” and further surgical excision was recommended for definitive classification. Later, the patient underwent right thyroid lobectomy and isthmusectomy.

Pathologic/ancillary findings

Gross examination of the specimen revealed two masses in the right thyroid lobe. The large mass (4.4 cm) was seen in the mid to lower aspect of the right lobe. The smaller mass (2 cm) was seen in the lower aspect of the right lobe. Isthmus was unremarkable. Sectioning of the masses into 3 mm slices showed a well-encapsulated homogeneous tan cut surface. The entire thyroid was submitted for microscopic evaluation, including the two thyroid masses.

**Figure 1:**
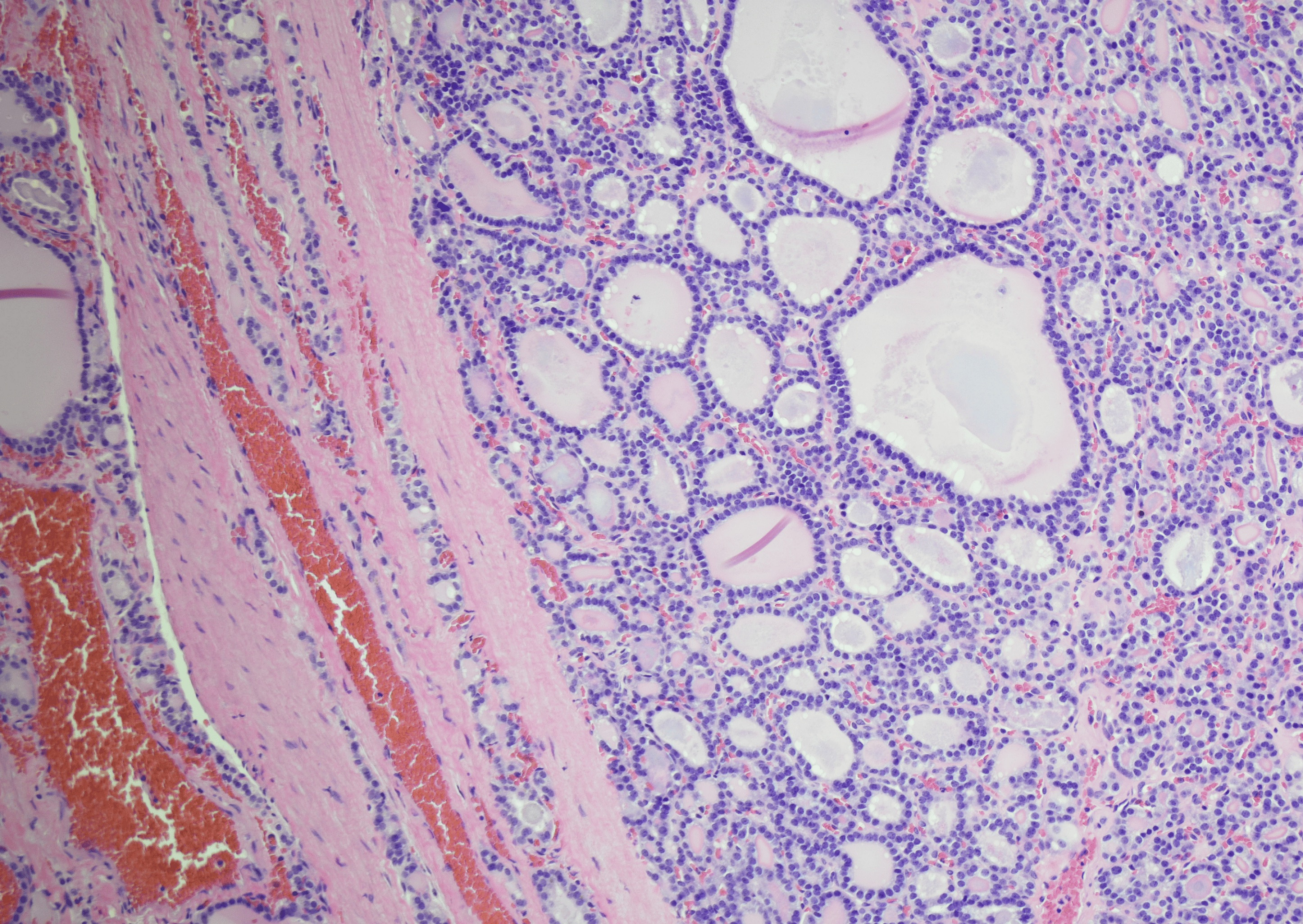
Low magnification (H and E, 10X) of the thyroid mass showing encapsulated neoplasm with predominantly microfollicular architecture.

**Figure 2:**
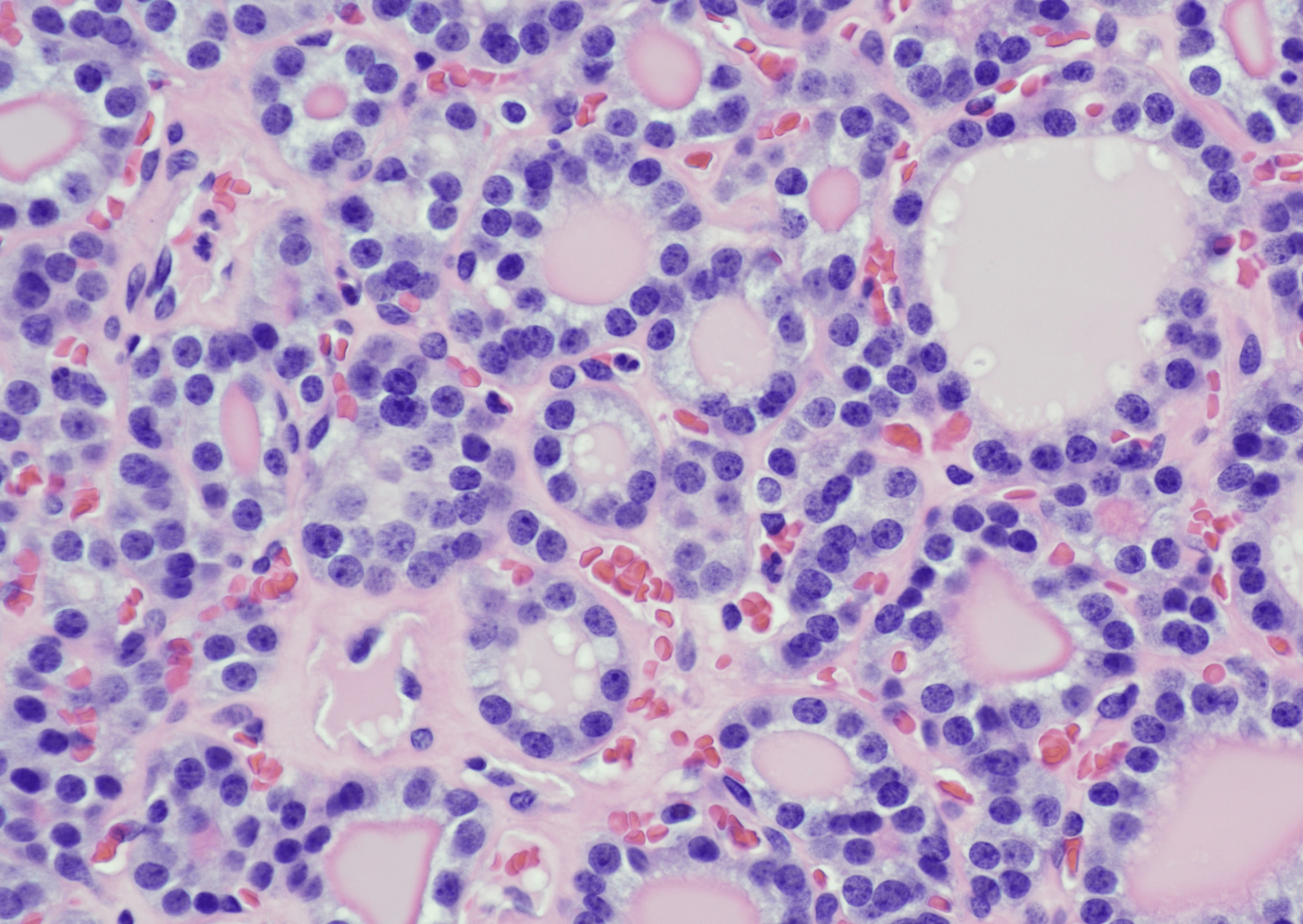
Higher magnification (H and E, 40X) of the thyroid mass showing small round hyperchromatic nuclei with the absence of nuclear features of papillary thyroid carcinoma.

The histologic examination showed encapsulated masses with a microfollicular pattern and intraluminal colloid (Figure 1). A compressed unremarkable thyroid was seen on the periphery. The lesional cells showed hyperchromatic small round basally located nuclei. Nuclear features of papillary carcinoma such as enlargement, grooves, inclusions, clearing, or nuclear contour irregularities were not seen (Figure 2). These findings were consistent with follicular adenomas.

**Figure 3:**
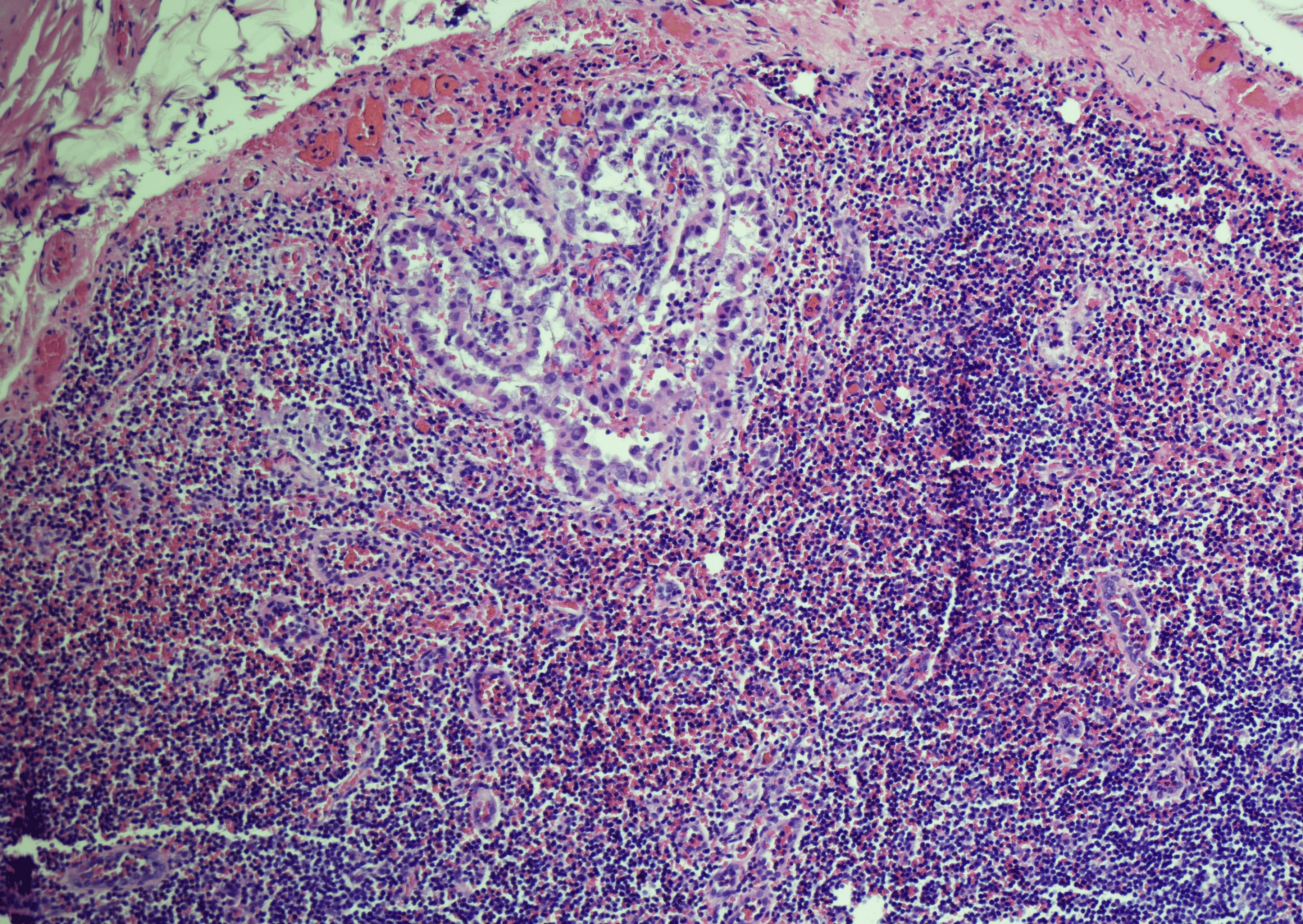
Low magnification (H and E, 10X) of the lymph node showing a cluster of epithelial cells in the subcapsular area of the lymph node.

**Figure 4:**
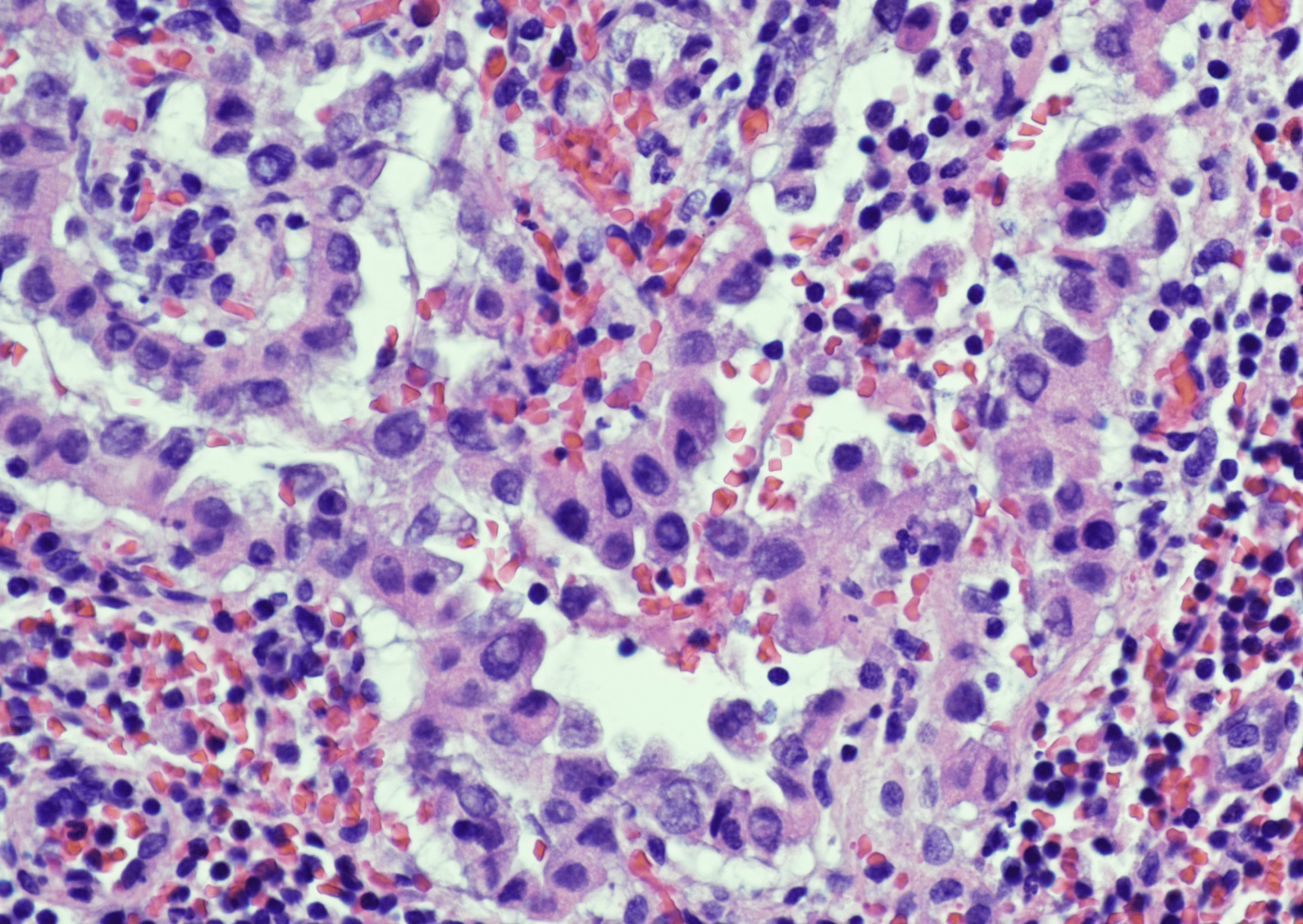
Higher magnification (H and E , 40X) of the lymph node showing the epithelial cells with nuclear enlargement, multiple nuclear pseudoinclusions, and grooves, consistent with metastatic papillary thyroid carcinoma.

**Figure 5:**
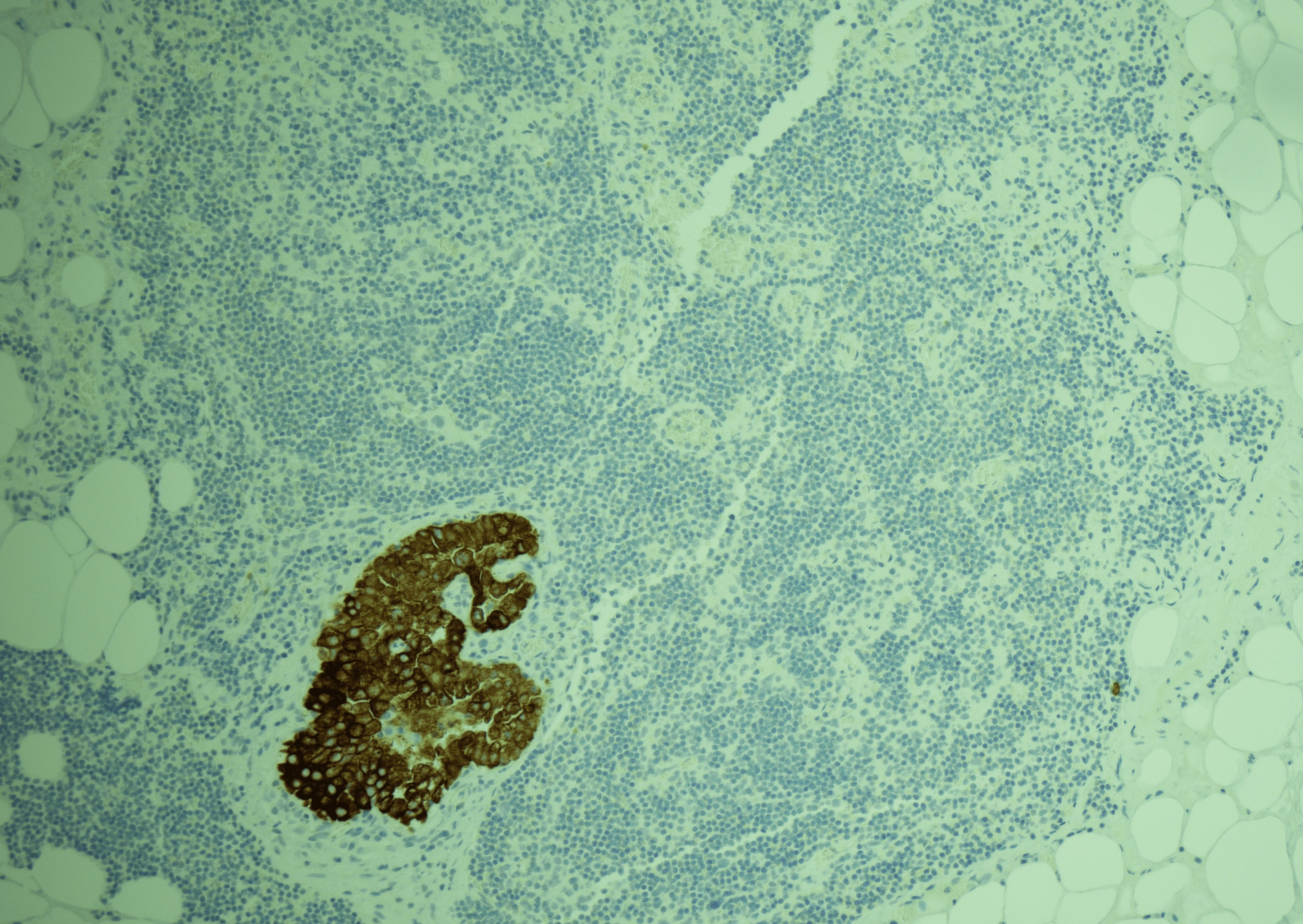
Low magnification (CK19 IHC, 10X) showing the tumor cells in the lymph node to be positive for CK19, confirming the morphologic impression of metastatic papillary thyroid carcinoma.

**Figure 6:**
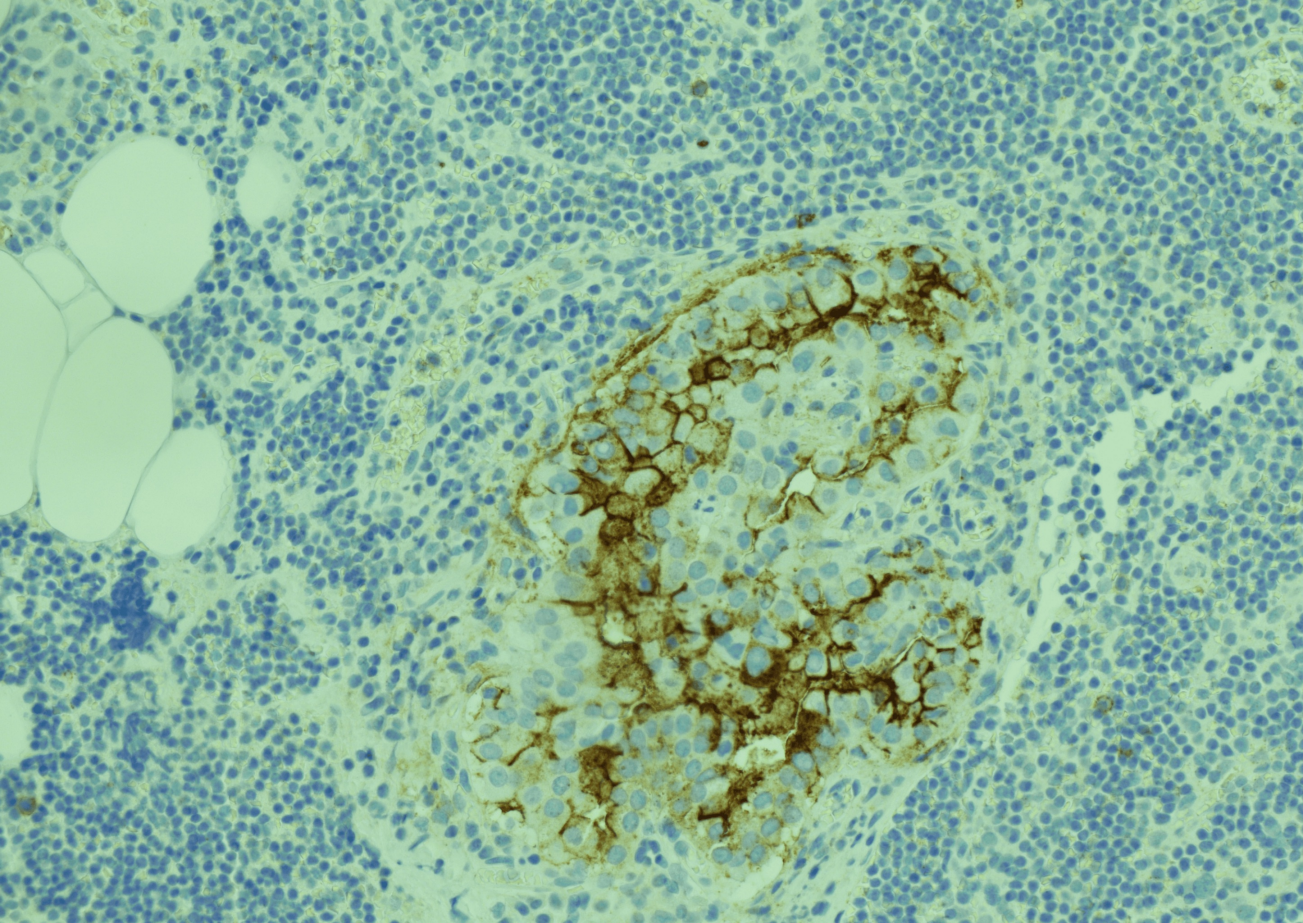
Medium magnification (HBME IHC, 20X) showing the tumor cells in the lymph node to be positive for HBME, confirming the morphologic impression of metastatic papillary thyroid carcinoma.

**Figure 7:**
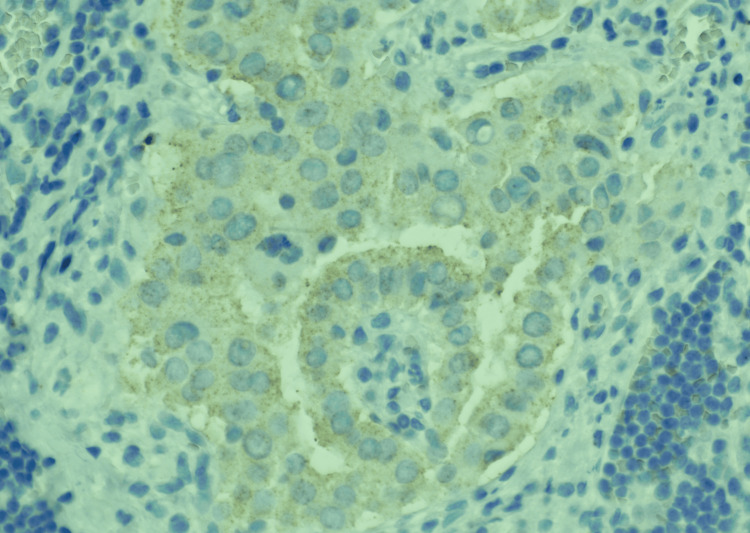
Higher magnification (BRAF IHC, 40X) showing the tumor cells in the lymph node with weak positivity for BRAF.

Interestingly, there were six lymph nodes in the perithyroidal fibroadipose tissue. Two of these lymph nodes had small foci of subcapsular epithelial clusters with nuclear enlargement, nuclear pseudoinclusions, and grooves, suspicious for metastatic PTC (Figures 3, 4). The immunohistochemical assay showed the atypical subcapsular epithelial clusters to be positive for CK19 (Figure 5) and HMBE-1 (Figure 6), confirming the diagnosis of metastatic PTC within the perithyroidal lymph nodes. One of two lymph nodes had faint BRAF V600 positivity (Figure 7).

There was no evidence of extra nodal extension. He was then treated with a 70 mCi ablation dose of I-131. The patient is followed up every year under his surveillance protocol and has showed no evidence of recurrence or metastasis so far.

## Discussion

Most of the thyroid cancers are asymptomatic and are typically seen as an incidental finding on ultrasound studies of the neck for unrelated reasons. They may present with palpable thyroid masses/nodules. Less often, thyroid cancers may present as recurrent laryngeal nerve palsy or parapharyngeal mass and rarely may present as distant or locoregional lymph node metastasis, as seen in our case.

There are a few reported cases of patients with isolated lymph node metastasis from PTC [3-5]. Several possible hypotheses could explain why a PTC would present metastatic to a lymph node without a primary tumor in the thyroid gland. Some of the theories that could explain these phenomena include primary tumor spontaneous regression, PTC in ectopic thyroid tissue, or a missed microscopic focus during histologic examination.

Spontaneous regression is an extremely rare phenomenon; however, there are reported cases where PTC regressed without treatment [2,6]. Possible mechanisms for regression that have been proposed include immune mediation, growth factor and cytokine-mediated mechanism, and hormonal mediation [6]. Literature also proposed that an asymptomatic tumor with minimal/no growth and that may never have posed a problem to the patient may regress spontaneously [2].

Another hypothesis is the presence of PTC in ectopic thyroid tissue. Ectopic thyroid tissue in general is unusual, found in 7%-10% of autopsies, and the development of carcinoma within ectopic tissue is exceedingly rare (1% of cases) [7,8]. Most ectopic tissue ends up located in the cervical midline (base of the tongue to the mediastinum), but the remainder of ectopic cases can present elsewhere, such as the anterior tongue, esophagus, diaphragm, and even duodenum [7,8]. Notably, our patient has an absent left thyroid lobe, indicating that while this could be a congenital absence (as per clinical notes), he may have thyroid tissue that is ectopically located.

Of course, there is always the likelihood that a focus of microcarcinoma of the thyroid was not captured during histopathologic evaluation of the submitted sections, even after meticulous and extensive sampling of the entire thyroid [2]. This is a reasonable theory, as in patients with microcarcinoma, the incidence of metastasis to the cervical lymph nodes can vary anywhere from 29% to 65% [9]. It is recommended that when grossing a thyroid, it is necessary to section the specimen into 2-3 mm slices and transilluminate the slices to identify any white or scarred areas that could be foci of microcarcinomas [10].

## Conclusions

Metastatic PTC presenting in the lymph nodes without a primary tumor in the thyroid is unusual. Careful examination of lymph nodes is crucial in ensuring a malignant diagnosis is not missed, as metastasis to a lymph node is indicative of a tumor’s capacity for lymphatic spread. It does have management implications. The prognosis of such cases depends on the extent of metastasis. Usually, a more favorable outcome is seen in cases where the tumor is confined to the lymph node compared to cases where the tumor presents with extra nodal extension.
